# ISS-An Electronic Syndromic Surveillance System for Infectious Disease in Rural China

**DOI:** 10.1371/journal.pone.0062749

**Published:** 2013-04-23

**Authors:** Weirong Yan, Lars Palm, Xin Lu, Shaofa Nie, Biao Xu, Qi Zhao, Tao Tao, Liwei Cheng, Li Tan, Hengjin Dong, Vinod K. Diwan

**Affiliations:** 1 Division of Global Health (IHCAR), Department of Public Health Sciences, Karolinska Institutet, Stockholm, Sweden; 2 Future Position X (FPX), Gävle, Sweden; 3 Department of Epidemiology and Biostatistics, School of Public Health, Tongji Medical College of Huazhong University of Science and Technology, Wuhan, China; 4 Department of Epidemiology, School of Public Health, Fudan University, Shanghai, China; 5 Institute of Public Health, Heidelberg University, Heidelberg, Germany; 6 Department of Sociology, Stockholm University, Stockholm, Sweden; 7 Department of Information Systems and Management, National University of DefenseTechnology, Changsha, China; 8 Center for Health Policy Studies, School of Public Health, Zhejiang University School of Medicine, Hangzhou, China; Northeastern University, United States of America

## Abstract

**Background:**

syndromic surveillance system has great advantages in promoting the early detection of epidemics and reducing the necessities of disease confirmation, and it is especially effective for surveillance in resource poor settings. However, most current syndromic surveillance systems are established in developed countries, and there are very few reports on the development of an electronic syndromic surveillance system in resource-constrained settings.

**Objective:**

this study describes the design and pilot implementation of an electronic surveillance system (ISS) for the early detection of infectious disease epidemics in rural China, complementing the conventional case report surveillance system.

**Methods:**

ISS was developed based on an existing platform ‘Crisis Information Sharing Platform’ (CRISP), combining with modern communication and GIS technology. ISS has four interconnected functions: 1) work group and communication group; 2) data source and collection; 3) data visualization; and 4) outbreak detection and alerting.

**Results:**

As of Jan. 31^st^ 2012, ISS has been installed and pilot tested for six months in four counties in rural China. 95 health facilities, 14 pharmacies and 24 primary schools participated in the pilot study, entering respectively 74256, 79701, and 2330 daily records into the central database. More than 90% of surveillance units at the study sites are able to send daily information into the system. In the paper, we also presented the pilot data from health facilities in the two counties, which showed the ISS system had the potential to identify the change of disease patterns at the community level.

**Conclusions:**

The ISS platform may facilitate the early detection of infectious disease epidemic as it provides near real-time syndromic data collection, interactive visualization, and automated aberration detection. However, several constraints and challenges were encountered during the pilot implementation of ISS in rural China.

## Introduction

Infectious diseases have for centuries ranked with wars and famine as major challenges to human progress and survival [1]. The emergence of ‘new’ infectious diseases, and the reemergence of ‘old’ infectious diseases, now present global problems. Preparedness for known and unknown infectious diseases is a top priority for public health systems. The sooner we can detect emergent public health events, the sooner we can take prompt actions against them. Therefore, in order to mitigate potential damages, early warning of infectious disease epidemics has long been one of the fundamental tasks for infectious diseases surveillance and a primary concern for public health institutions.

Syndromic surveillance system, which collects non-specific syndromes in the early stages of disease development, has great advantages in promoting the early detection of epidemics and reducing the necessity of disease confirmation. By identifying abnormal aggregation of health related events, early signals of outbreaks may be detected [2], [3], [4]. In 2003, over 100 different US health jurisdictions used syndromic surveillance systems to augment their public health surveillance [5], such as the ESSENCEα [6], Real-time Outbreak and Disease Surveillance (RODS) [7], Light Weight Epidemiology Advanced Detection and Emergency Response System (LEADERS) [8]and so on. In recent years, more and more syndromic surveillance systems have been established for the early warning of natural diseases outbreak as well as bioterrorism events [9], [10], [11]. In Asian countries, Japan, Korea and Taiwan (in China) also developed syndromic surveillance systems for diseases outbreak detection [12], [13], [14].

Syndromic surveillance systems have been mostly established in developed countries or areas where modern information technology infrastructure is well established and professional human resource support is available. However, syndromic surveillance approach also holds promise to augment public health surveillance in resource constraint settings. It offers: (a) immediate reporting where laboratory confirmation is not possible or impractical; (b) a simple and stable case definition as it reports what is actually seen; and (c) automated detection of abnormal disease clusters by using advanced mathematical algorithms with daily surveillance data [15], [16], [17]. Until the current study, there are no public reports on the development and implementation of a syndromic surveillance system in rural China.

In 2010, European Commission started to fund a FP7 project on developing a syndromic surveillance system in rural China to complement the existing case reporting system (ISSC: 241900). With reference to the ideas and experiences in the development of Real-time Outbreak and Disease Surveillance (RODS) and Electronic Surveillance System for the Early Notification of Community-Based Epidemics (ESSENCE II), a new web-based syndromic surveillance system was developed (Website: isscproject.com), combining with modern communication and GIS technology. So far, this system has been installed and pilot tested in four counties in rural China. This paper will describe key features and potential applications of the ISSC product in rural China or other similar resource limited settings.

## Methods

### FPX and CRISP

Future Position X (FPX) is a leading cluster for innovative and expanding use of Geographical Information in Europe. In the ISSC project, FPX has entered into a robust collaboration with two institutions in Europe, Karolinska Institutet and Heidelberg University, and two universities in China, Huazhong University of Science and Technology and Fudan University to create a web-based syndromic surveillance system (ISS) in rural China. Before the development of ISS, FPX has established a ‘Crisis Information Sharing Platform’ (CRISP), which has the potential to be adapted and modified into ISS.

CRISP is originally a development project aimed to help emergency services by streamlining real-time information to facilitate decision-making. The platform is based on Google Maps as a background map with dynamic layers from other systems. CRISP is capable of 1) facilitating the collection of information from many different sources by combining and visualizing data in a customizable interface; 2) aggregating information by topic or event, making it easier for users to quickly interpret and identify important information links; 3) delivering emergency messages customized to each recipient's profile; and 4) supporting shared situational awareness through integrated geographic information. Most of these functions within CRISP can be applied in ISS with modifications.

CRISP is based on an open architecture, which facilitates further development of additional applications on the existing platform. The platform is built using PHP 5.3.2 as the programming language and MySQL as the database manager.

### Structure of ISS

**Figure 1:**
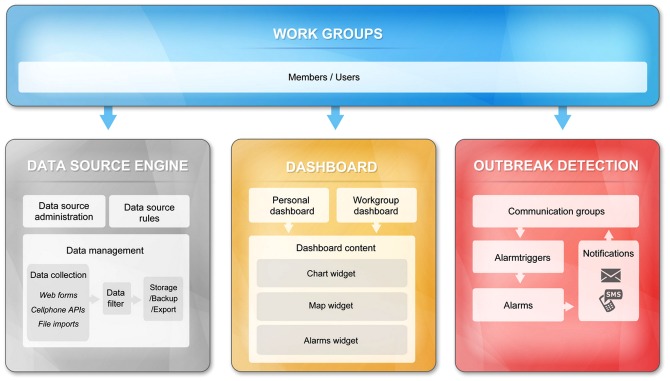
Structure of ISS platform. ISS has four interconnected components: 1) work group and communication group; 2) data source and collection; 3) data visualization; and 4) outbreak detection and alerting.

ISS has four interconnected components (see Figure 1): 1) work group and communication group; 2) data source and collection; 3) data visualization; and 4) outbreak detection and alerting. Within each component, ISS offers users a variety of tools and options so as to create a customized and scalable electronic disease surveillance system according to users’ needs and capabilities. For example, users can decide the information they would like to collect or display by editing web forms, and personalize the setting of parameters and thresholds of models by editing ‘alert triggers’. Besides, ISS has an important module of language translation, which makes the platform has the ability of being transformed into any other languages by users. As of this writing, the system already has English and Chinese versions.

#### Work groups and Communication groups

The *work group* and *communication group* module enables the system administrators to restrain data accessibilities to increase data security and privacy protection, yet by the same time facilitate the sharing of valuable information among authorized users.

Similar to other syndromic surveillance systems, ISS has a password-protected, encrypted Web site in which users can input, review, query, export, analyze and visualize surveillance data. When a user logs in, the system will check the user’s profile and only allow the user’s activities within his/her predefined authorities. System administrators have the right to create structural work groups, invite new users to different work groups and define authorities of different group members. For example, we established a structure of ‘Country’-‘Province’-‘City’-‘County’-‘Town’-‘Village’ and each user has restrained rights to work on the data within his or her jurisdiction.

Sharing of individual patient data across different work groups or users is generally not helpful, however, once these data have been transformed into meaningful information, for example, alert information, it will be valuable to share that information with others [18]. In the ISS platform, system administrators also can create communication groups and define group members for a specific communication group. Users in the same communication group can share information specifically belongs to that group. For example, they can set several alert triggers, and receive notifications of alerts generated from predefined alert triggers by email. The composition of a communication group is flexible. It may include one or more work groups, or a subset of group members not necessarily a whole work group.

#### Data Sources and Collection

**Figure 2:**
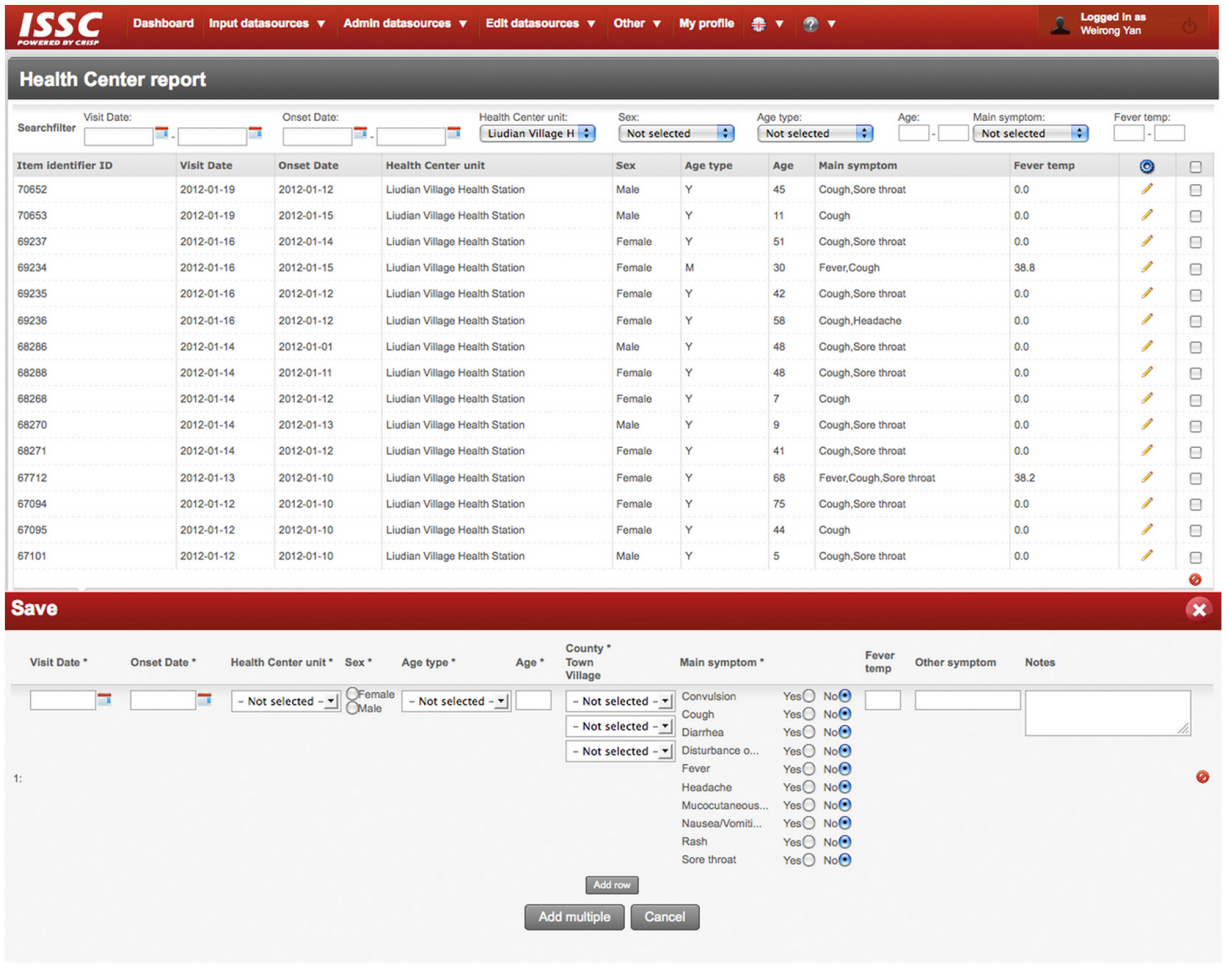
Web-form for data collection in health center report. The web-form for data collection in the ISS platform is very simple for users to fill in and it usually takes less than one minute for a familiar user to input one case. High-level users can also edit the web-form and define different checking rules for data input.

ISS collects daily syndrome information from three different data sources, including main symptoms of patients who present at health facilities, medication sales from retail pharmacies and primary school absenteeism. An example of interface for health center data report is shown in Figure 2, and more detailed description of these data sources can be found in a published study protocol [19]. Data records are standardized and can be reported by a data collector either directly through filling of web forms by computer, or importing into the system via Excel files. Data collectors include nurses or doctors in health care units, staff in retail pharmacies and teachers in primary schools. If data collectors have no access to Internet, they will inform local CDC staff, who then is responsible to assist the data report.

In ISS, system administrators are able to set different checking rules for data input. Once the data is reported and transferred into the central database, the system checks for logical erroneous and missing values, such as an onset date of symptoms is later than the patient’s visit date, the age value fall outside of reliable limits, body temperature is missing when the symptom of ‘fever’ is chosen, etc. Once the system detects any errors or missing, it will display a popup message on the web page, indicating that the procession of data transfer is failed and the reason for the failure. After having corrected the errors, data collectors submit the web form again to transfer data.

#### Data visualization

**Figure 3:**
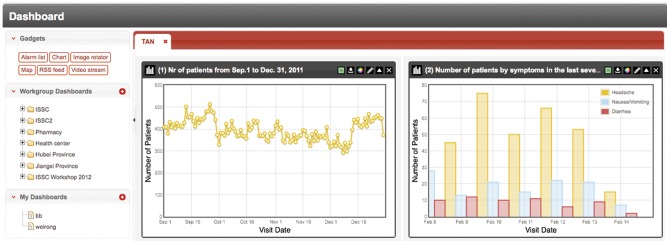
Chart examples in the dashboard. Through the ISS platform, users have the ability to show the characteristics of data in the format of lines, bars and pies.

With the ISS platform, users can create a *dashboard* (Figure 3), which works as a desktop interface where users can add different widgets to present data in customized *alarm lists*, *map*s and *chart*s. *Dashboard* can be related to a specific workgroup (*workgroup dashboard*) or a single user (*personal dashboard*). A ‘*workgroup dashboard*’ contains data that is linked to a specific work group while ‘*personal dashboard*’ contains data from all the workgroups that the user belongs to.

*Alarm list* present all alerts information, which belongs to one or several communication groups chronologically. This function allows users to check the detailed information of each alert in an aggregate way.

*Map*s are created to present generated alerts geographically; this function is currently based on Google Map via the Internet. However it is planning to move to the electronic maps provided by the local institutions. This is mainly due to a real-time dynamic visualization in Maps requires a high-speed Internet connection while the Internet connection to Google Map in rural China is not good at present.

The *Chart* screen provides a general plotting capability, in which the characteristics of data can be displayed in the format of lines, bars and pies. In the widget box of *chart*, users can set the refresh rate, height and width of the chart. Users can also choose to view data for different data source (health facilities, pharmacies or primary schools), geographic region (province, county, town or village), and time interval (e.g., three days, one week, one month, etc.).

#### Outbreak detection and alerting

The *Alarm Trigger* module incorporates models to detect aberrations of syndromic observations from daily surveillance data. When an alert is triggered, the system will automatically send e-mail notifications to subscribed communication groups. At this stage, ISS has incorporated nine detection algorithms into the system, including models for temporal analysis (Shewhart Chart (P Chart), Moving Average (MA), Exponentially Weighted Moving Average (EWMA), Cumulative Sums (CUSUM)), spatial analysis (Recursive Least Square (RLS) Method, Small Area Regression and Testing (SMART), Bayesian spatial scan statistics), and spatial-temporal analysis (Space-time Scan Statistics and What is Strange About Recent Event (WSARE)). These models are currently widely used in other surveillance systems [20], [21], however, since there is a shortage of historical data in ISS and the system is applied in a very different setting, the applicability of these models yet need to be evaluated.

**Figure 4:**
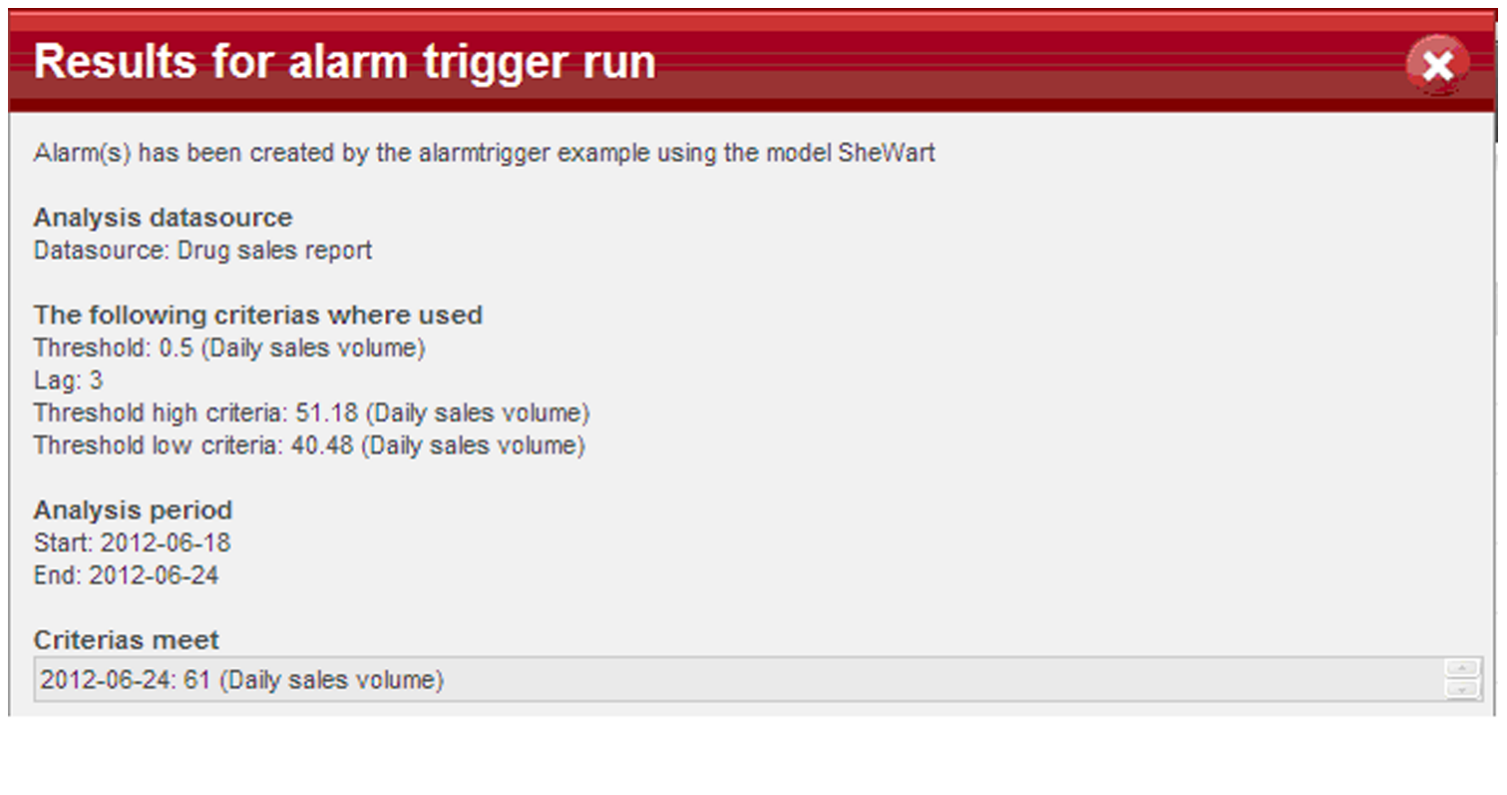
Running result of a detection model. Each time when the user activates an alarm trigger, a running log will be generated and saved in the system, including data sources, model parameters, and detection time if the model threshold is exceeded to generate alerts.

ISS platform enables flexible and customizable model settings. The system sets default values for model parameters and threshold, but it also allows users to modify the model settings based upon needs. They can choose to run models for a specific data source or time interval. They can also set specific time for the system to run models automatically, such as to run a model every 8 hours per day or at 6∶00 every morning, etc. Each time a detection model is run, a log will be generated and saved in the system, including data sources, model parameters, and detection time when the model threshold is exceeded to generate alerts (Figure 4).

**Figure 5:**
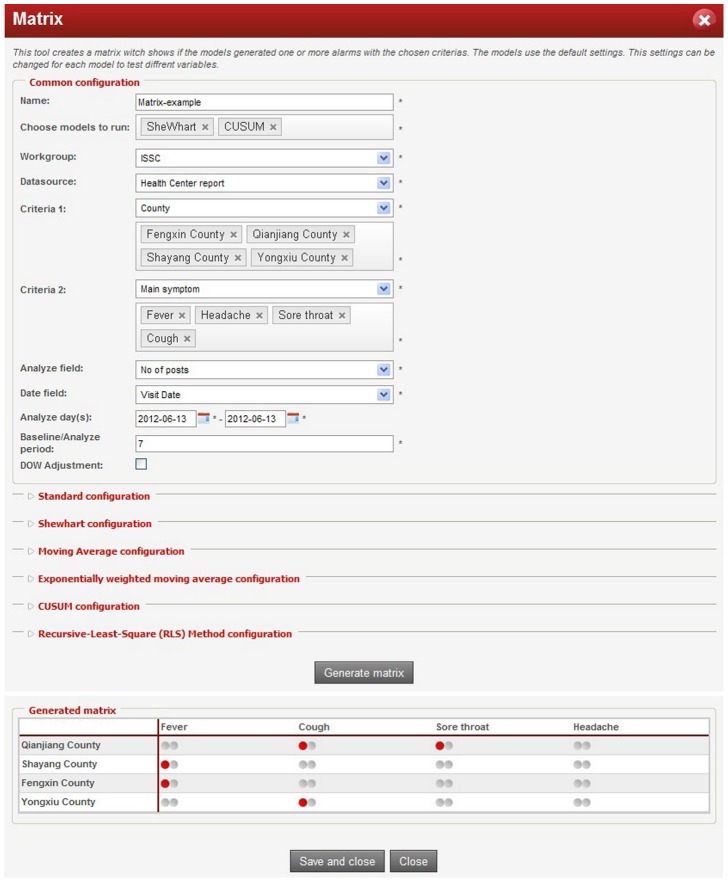
Generated matrix of selected models using data source from health centers. The generated matrix showed the running results of SheWhart and Cusum models when they were applied to the number of patients with four different symptoms in the four counties. Each point represents a model, and the red point represents there is one alert generated from this model based on the selected data source.

In order to get an overview of the running results and to evaluate different models, a comparison *matrix* is created when selecting more than one model to run using the same data source (Figure 5). Users can choose *criteria 1* and *criteria 2* to define the “row” and “column” of the *matrix*. For example, the setting of *criteria 1 = ’ county’,* and *criteria 2 = ’ main symptom’* will generate a *matrix M* in which each element *M_ij_* represents the result of detection models for symptom *j* in county *i*. When the *matrix* is generated, the users can click on each matrix element (small point in Figure 5) to check more information on the analysis of that model.

### Ethics Statement

The written consents were obtained from the local health authorities that they were willing to participate in the study and the ISS system can extract existing information from their clinic logs. All patient information was de-identified and only aggregated data was used for data analysis. The study has been ethically approved by the Institutional Review Board of Tongji Medical College and Fudan University.

## Results

After the system had been developed, six months was used as the period of pilot study to test how the system was functioning under a real life condition, during the workflow from data collection to alerts generation.

Two counties in Hubei (Qianjiang and Shayang) and Jiangxi Province (Fengxin and Yongxiu) respectively joined this project, among which two towns in each county in Jiangxi Province and one town in each county in Hubei Province joined the pilot study, and all village health stations under the administration of the participating towns were recruited into the study. So far, there have been 95 health facilities, 14 pharmacies and 24 primary schools transferring daily data to the ISS platform, generating respectively 74256, 79701, and 2330 daily records in the central database of ISS from Aug.1, 2011 to Jan.31, 2012.

### Data collection approaches used in the pilot study

Even computer and Internet use are increasing in rural China, electronic medical records are not available in most health departments [22]. Consequently, unlike developed settings using automated electronic data capture, ISS involved additional manual labor to type data into the system. In health facilities, the process of data collection begun with health personnel gathering information from paper-based forms, and then filling out the required data into web-based forms in ISS. If the health personnel have no access to Internet in some units, s/he needs to ask people at local CDC to transfer data via mobile phones, landlines (voice instruction), fax (paper copies), etc. The data collection approach in health facilities is currently similar as those used in other syndromic surveillance system established in resource-poor settings [23], [24]. However, the ISS system is scalable to automated data retrieving once electronic data source is available for using.

Due to the concerns on the security of business information, automated data retrieving from electronic sales system was not allowed by pharmacy owners in the pilot study. In big pharmacies, staff exported data from their electronic sales information system and saved the required data as Excel files, and then imported such files into the ISS system. In small pharmacies, staff usually logged into the system to fill out web forms for data collection as most of them didn’t have an electronic sales information system; the same collection approach was also used in primary schools.

Although SMS text messages by mobile phones was considered as an option for data transferring in the study protocol, we did not take this option in the field implementation. As more than 90% surveillance sites in these four counties were equipped with computers and Internet, the number of study sites without computer was becoming less. Additionally, local data collectors found it was very difficult and time-consuming to use SMS service to send error-free text messages to central database, as most of them were elderly and less educated.

### Training and supervision over data collectors

With the usage of computer and Internet rapidly spreading in rural China, we found it was no problem for most data collectors to use the ISS platform for data input, review, query, etc. However, due to the lack of computer operation skills, it was difficult for some doctors, particularly the old village doctors, to input data into the ISS system through web forms. Some village doctors quickly became familiar with the ISS system, while the others needed a focused and repeated training. As of January 2012, more than 90% of surveillance units at the pilot study sites were able to send daily information into the system.

**Figure 6:**
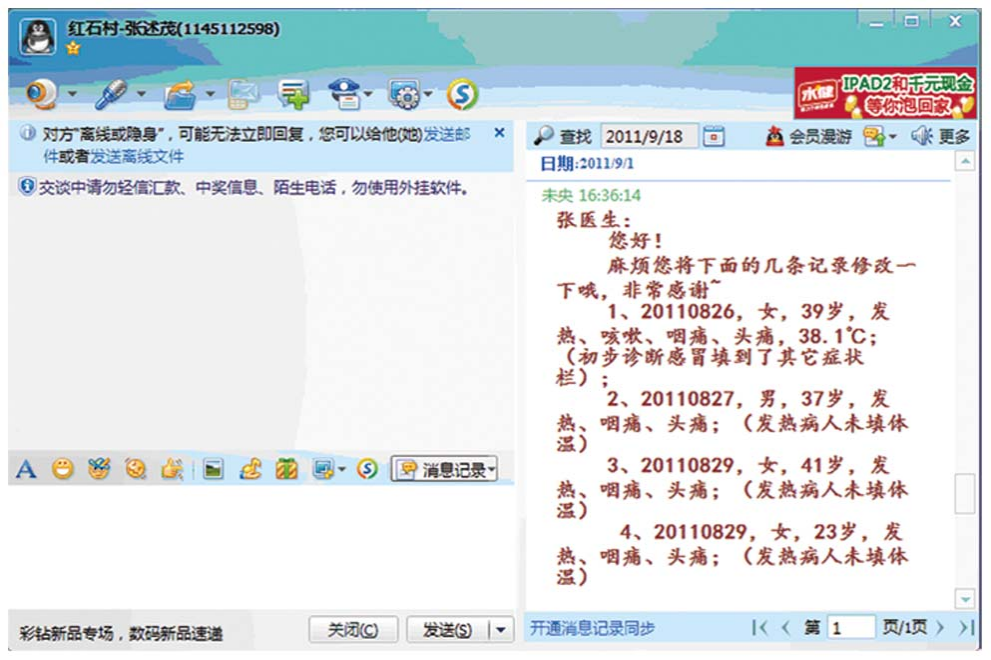
A system supervisor reminding a data collector to make corrections via QQ messenger in Chinese. QQ messenger is popularly used for online communications in China. In the pilot study, we used QQ to create a communication platform for data quality control, like reminding a timely data reporting, correcting reporting mistakes, etc.

System supervision personnel, consisting of researchers from the two Chinese participating institutions and staff for disease surveillance at local CDCs, need to check any missing or erroneous data reporting frequently. The supervision personnel at local CDCs will make a phone call to or an online communication with the surveillance units to verify successful data transfer if there were no data input in two consecutive days. During the daily supervision, the most popular instant messenger used in China, QQ [website: www.qq.com], was used to create a forum for communications between supervisors and data collectors, e.g., remind data collectors to report, add missing data, correct reporting mistakes (Figure 6), etc. In order to check the compliance of data collectors, help to correct erroneous data and refresh the training to data collectors, system supervision personnel had visited study sites several times during the pilot study.

### Overview of the pilot data

As an illustration on the outcomes of the pilot study, we present in this section the symptomatic data collected from health facilities in Shayang and Qianjiang County, Hubei province.

From Aug.1, 2011 to Jan.31, 2012, one county hospital, one township hospital and 16 village health stations in Shayang, and one county hospital, one township hospital and 20 village health stations in Qianjiang joined the pilot study. By the end of January, 2012, the number of patients with at least one targeted symptoms (PTS) recorded in the system was 9958 and 19641 for Shayang and Qianjiang, respectively. It was worth noting that village health stations accounted a major fraction of the data source, with 7889 records (79.22%) in Shayang and 17170 records (87.42%) in Qianjiang, respectively. The ability of collecting such amount of syndromic information from village level which would otherwise be missed indicated the feasibility and potential application of the ISS system for real-time syndromic surveillance in rural China.

The age distributions of PTS in Qianjiang and Shayang were similar (Table 1). Among all PTS visiting rural health facilities, patients aged over 45 years old or under 15 years old accounted for relatively larger proportions. Young adults between 15 to 34 years old were less frequent visitors to rural health centers because of the targeted symptoms. According to the sixth population census data, the population of Qianjiang was composed of 12.98% from 0–14, 77.65% from 15–64, and 9.37% for 65 or older [25], and the population in Shayang County also had a very similar age distribution [26]. The proportion of 0–14 years group was much higher among PTS in comparison to the census data, indicating that this group was at a higher risk for infectious diseases, especially respiratory and gastrointestinal infectious diseases. The proportion of group aged 65 years old or older was also higher among PTS compared to the census data. In all, these age distributions suggested that the ISS system was able to capture age groups at higher risk for infectious disease.

**Table 1:** Demographic characteristics of PTS in Qianjiang and Shayang counties during the pilot study (Aug.1, 2011– Jan.31, 2012).

	Qianjiang County		Shayang County	
Age groups(years)	Number of patients	Percentage (%)	Number of patients	Percentage (%)
<5	2913	14.83	1677	16.84
5-	2325	11.84	1423	14.29
15-	1267	6.45	416	4.18
25-	1723	8.77	602	6.05
35-	2766	14.08	1170	11.75
45-	3220	16.39	1655	16.22
55-	2944	14.99	1550	15.57
65-	2483	12.64	1465	14.71
Gender				
Males	10038	51.11	4818	48.38
Females	9603	48.89	5140	51.62

Among the ten targeted symptoms, cough was the most frequent symptom during the pilot study period, accounting for 43–44% of the records (Table 2). Other common symptoms are sore throat, fever, and headache, which were observed in more than 10% of the patients. Symptoms of rash, mucocutaneous hemorrhage, convulsion and disturbance of consciousness were rare. The frequency distribution of the ten symptoms was similar in these two counties, except for fever and headache. The symptoms of diarrhea and vomiting/nausea were not very common, which may due to the specific surveillance seasons (from Aug.1, 2011 to Jan.31, 2012), as the local epidemic season of gastrointestinal infectious disease is usually from May to October in Hubei Province.

**Table 2:** Distribution of the ten targeted symptoms in Qianjiang and Shayang County during the pilot study* (from Aug.1, 2011 to Jan.31, 2012).

	Qianjiang County		Shayang County	
Symptom	Number of patients	Percentage	Number of patients	Percentage
Cough	13331	43.68%	6979	43.49%
Sore throat	7438	24.37%	3488	21.74%
Fever	3163	10.36%	2449	15.26%
Headache	4662	15.27%	2088	13.01%
Diarrhea	1003	3.29%	487	3.04%
Vomiting/Nausea	724	2.37%	307	1.91%
Rash	116	0.38%	231	1.44%
Mucocutaneous hemorrhage	66	0.22%	11	0.07%
Convulsion	13	0.04%	5	0.03%
Disturbance of consciousness	7	0.02%	1	0.01%

* : There were patients with more than one targeted symptoms.

**Figure 7:**
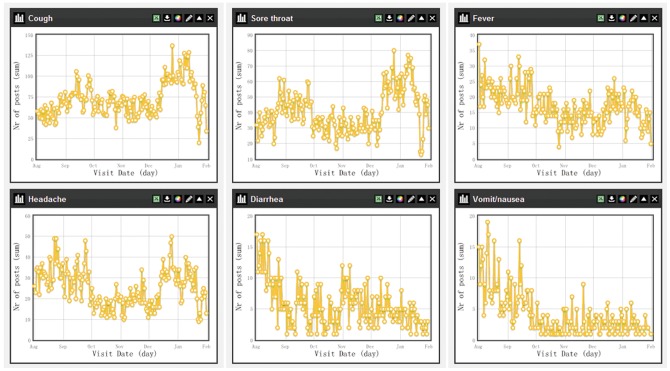
Time series plots of six main symptoms in Qianjiang County (Aug.1, 2011–Jan.31, 2012). The time series plots of the main symptoms in Qianjiang County was a screenshot from the *Chart* of the *Dashboard* in the ISS system, which provided users a preliminary description and analysis of rough data on daily numbers of each symptom in the county.

**Figure 8:**
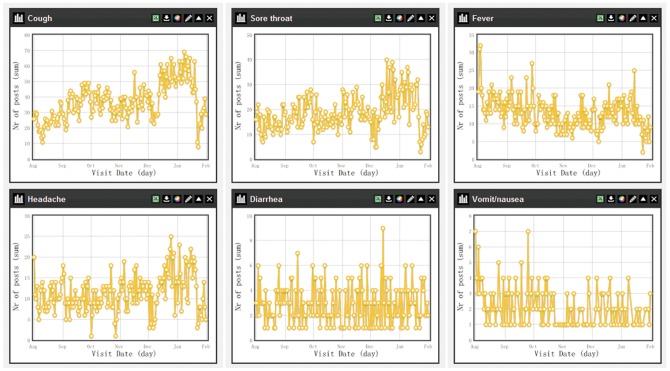
Time series plots of six main symptoms in Shayang County (Aug.1, 2011–Jan.31, 2012). The time series plots of the main symptoms in Shayang County was a screenshot from the *Chart* of the *Dashboard* in the ISS system, which provided users a preliminary description and analysis of rough data on daily numbers of each symptom in the county.

The time series plots of daily number of each specific symptom can be visualized through the Dashboard in the ISS system, see examples in Figure 7 and Figure 8. During December and January, there were obvious increases in the daily numbers of visits for the symptoms of cough, sore throat and headache in both Qianjiang and Shayang County, which may indicate that more respiratory infections occurred because of the cold weather. However, the daily number of the visits for fever didn’t increase as much as these three symptoms. A steep decrease was found in the daily number of visits for the four major symptoms (i.e., cough, sore throat, fever and headache) on Jan. 23, which was the first day of Chinese Lunar New Year in 2012. Many Chinese people are reluctant to visit doctors during Lunar New Year for mild diseases as it is believed to bring misfortune, and therefore many rural health facilities are closed up during that time. In Qianjiang County, the visits of diarrhea and vomit/nausea decreased from October, which was in concordant with the local epidemic season (from May to October) of gastrointestinal infectious disease.

## Discussion

With China’s economic development, the government is increasingly attaching great importance on the establishment of early warning system of disease outbreak. With the strong political support we see a window of opportunity to develop and implement a syndromic surveillance system in rural China. However, several constraints and challenges were found from the pilot study.

In most developed settings, syndromic data is automatically retrieved from electronic systems, encrypted, archived, and processed at a secure facility, combing free text and structured electronic medical record entries [12], [27], [28],[29],[30]. Although computer and Internet use are rapidly increasing in rural China, medical records of patients’ main symptoms or chief complaints, are paper-based in most health facilities [22]. Consequently, manual labor is needed to collect the syndromic data from paper-based forms and then enter into the web-based system. The manual collection approach increases the demand for additional labor and human intervention, and finally results in decreasing compliance of data collectors and increasing the difficulties of data quality control. This is one of the biggest challenges for developing a syndromic surveillance system in resource constraint settings. The completeness, timeliness and accuracy of data collection might be damaged due to additional amount of human labor involvement, which might eventually harm the performance and sustainability of the ISS system.

There are different types of data stream, which are potentially to be integrated in syndromic surveillance, and therefore it is imperative to develop a multi-sector cooperation and data sharing strategy between different data streams owners. In the current study, we found that some surveillance units, such as pharmacies, were reluctant to provide data as they had little collaboration and data sharing with public health agencies. The other related stakeholders, like health care facilities, food and drug administration (FDA), Education Administrative Department, and Quarantine Bureau also didn’t have an official data sharing policy with public health agencies [31].

The poor infrastructure of information and electricity network in rural areas also constraints the development of a web-based syndromic surveillance system. Power outages and instable Internet connection, all increased data reporting difficulties in rural China. Additionally, a considerable portion of surveillance units was lacking of basic computer maintenance skills, which influences the data reporting once there is any problem with the computer.

Despite the challenges regarding data collection and sharing, the ISS system has significant advantages over the conventional surveillance system. The data reporting procedure was very simple and flexible. Even for data providers with little computer knowledge, they can practice and become skilled in a very short time. The preliminary data analysis showed the ISS system had the potential for identifying the change of disease patterns within the community. We observed from the pilot data a few patterns which were consistent with the seasonal epidemics in the local setting, e.g., the increase of respiratory related symptoms in December, the decrease of gastrointestinal related symptoms in October, and the rapidly declined number of patients during the Spring Festival, etc. The analysis of the six month pilot data described in this paper primarily aimed to demonstrate the feasibility of collecting functional data from surveillance sites through the platform. Further validation of the ISS data will be required to formally assess its capability to early detection of public health incidents, which needs a longer time series of data in formal implementation with more surveillance units involved.

### Summary

By combining with modern information technology, the ISSC project is intended to develop a syndromic surveillance system to complement the traditional case reporting system in rural China for the early detection of outbreaks. Although at this stage, it is hard to determine if the system is feasible and sustainable in a long run, the project will provide knowledge and experience on developing and implementing a web-based syndromic surveillance system in rural China or other similar settings. With the growing availability of electronic data sources, the system can be scalable to achieve automated data fetching with electronic data, which can decrease the demand of manual labor for data collection and reduce data report error, making the system more sustainable and timely responsive.
